# Dr. Bardsley's Case of Hydrophobia Simulata, with Remarks

**Published:** 1826-07

**Authors:** 


### HYDROPHOBIA SIMULATA.

In the second number of the New Edinburgh Journal there is a case related by Dr. Bardsley of Manchester, where a female servant, without any ostensible object, applied some irritating substance to one of her legs, asserted that she had been bitten by a suspicious dog, and afterwards exhibited a train of hysterical symptoms, some of which assimilated with certain phenomena attendant on hydrophobia. We agree with Dr. Bardsley that this young female produced an artificial sore—that she afterwards renewed it in the hospital—that she attempted to deceive the medical officers there on two or three occasions—and that, shortly after her discharge, she committed a crime which sent her to a prison:—and yet, we are inclined to think that all this was not simulation, in the common sense of the word, but a species of mental aberration in a nervous female, which it was not in her power to resist. Dr. Beck observes that "diseases are generally feigned from one of three causes—fear, shame, or the hope of gain." Yet Dr. Bardsley admits that his patient "must have been influenced by some other motive; for she had nothing to fear, nothing to be ashamed of, and no prospect of gain." "He can only attribute her disgraceful conduct to that same depravity of heart, and want of moral principle, which led her to the commission of a more heinous crime, shortly after her discharge from the hospital." But we would submit to Dr. Bardsley, whether the following phenomena were simulated?—"Pulse was rapid and strong—tongue furred—skin dry—bowels constipated—*catamenia absent for several months.*" p. 250. We are led to a more charitable conclusion. We believe the poor girl was actually ill—that her nervous system was deranged—and that, under a partial hallucination which she could not resist, she was led blindly by a predominating impulse to acts which, in a state of perfect corporeal sanity, she would have abhorred. There is a young lady now in London, affected with some cardiac disorder, and of a highly hysterical disposition. She has been long attended by our friend Dr. Nuttall, whom we have often met in consultation on the case. Sometimes, in the midst of the most calm and intelligent conversation, she will start from her sofa, like a tygress, and throttle her medical attendant, or pummel him soundly with clenched fists—and then as suddenly fall back exhausted and pour forth a flood of tears at the thought of what she had done. Is not this a momentary obscuration of the intellect—a blind and involuntary impulse which she has no power to resist—no previous wish to put in force—no object thereby to obtain? We think so. And we conscientiously believe that such was the situation of Dr. Bardsley's unfortunate patient. The strong arm of the law, however, will make no such allowance for her last act—and the event, by curing her of her nervous and hysterical disorder, will, we have no doubt, put an end to her supposed "simulations," as well as to her propensity to crime.

